# Synthesis and preliminary structure-activity relationship study of 2-aryl-2*H*-pyrazolo[4,3-*c*]quinolin-3-ones as potential checkpoint kinase 1 (Chk1) inhibitors

**DOI:** 10.1080/14756366.2017.1404592

**Published:** 2017-12-06

**Authors:** Ivana Malvacio, Alberto Cuzzolin, Mattia Sturlese, D. Mariano A. Vera, E. Laura Moyano, Stefano Moro

**Affiliations:** a Department of Organic Chemistry, INFIQC, School of Chemical Sciences, National University of Cordoba, Cordoba, Argentina;; b Molecular Modeling Section (MMS), Dipartimento di Scienze Farmaceutiche, Università degli Studi di Padova, via Marzolo, Padova, Italy;; c Department of Chemistry, QUIAMM-INBIOTEC, School of Exact and Natural Sciences, National University of Mar del Plata, Mar del Plata, Buenos Aires, Argentina

**Keywords:** 2-Aryl-2*H*-pyrazolo[4,3-*c*]quinolin-3-one (PQ), serine-threonine checkpoint kinase 1 (Chk1), molecular docking, molecular dynamics (MD), supervised molecular dynamics (SuMD)

## Abstract

The serine-threonine checkpoint kinase 1 (Chk1) plays a critical role in the cell cycle arrest in response to DNA damage. In the last decade, Chk1 inhibitors have emerged as a novel therapeutic strategy to potentiate the anti-tumour efficacy of cytotoxic chemotherapeutic agents. In the search for new Chk1 inhibitors, a congeneric series of 2-aryl-2 *H*-pyrazolo[4,3-*c*]quinolin-3-one (PQ) was evaluated by *in-vitro* and *in-silico* approaches for the first time. A total of 30 PQ structures were synthesised in good to excellent yields using conventional or microwave heating, highlighting that 14 of them are new chemical entities. Noteworthy, in this preliminary study two compounds **4e_2_** and **4h_2_** have shown a modest but significant reduction in the basal activity of the Chk1 kinase. Starting from these preliminary results, we have designed the second generation of analogous in this class and further studies are in progress in our laboratories.

## Introduction

The pyrazolo[4,3-*c*]quinolone nucleus has a broad variety of pharmacological activity, making it an attractive structure for synthesis in recent years. Such heterocyclic system has been incorporated in ligands of the γ-aminobutyric acid type A receptor (GABA_A_) or benzodiazepine receptor. One of the most interesting properties of these ligands is the change of the intrinsic activity caused by small structural modifications. Their activity can shift from agonist (anxiolytic, hypnotic, anticonvulsant), through antagonist (without pharmacological response) to inverse agonist (pro-convulsant and anxiogenic)^1–4^. The pyrazolo[4,3-*c*]-quinolone have also been identified as cyclooxygenase-2 (COX-2) inhibitors which can reduce pain and inflammation^5^, as phosphodiesterase-4 inhibitors with antiasthmatic properties^6^, as well as Topoisomerase II inhibitors, which results in a high level of cytotoxicity and antitumour activity^7^^,^^8^.

During the last decade, heterocycles sharing a common pattern with the previously studied pyrazoloquinolones have emerged as checkpoint kinase 1 (Chk1) inhibitors^9–11^. The serine-threonine protein kinase Chk1 is a key mediator in response to DNA damage which, along with the tumour suppressor protein p53, is required for cell cycle arrest and activation of DNA repair before progressing into mitosis. However, many tumour cells rely only on Chk1 checkpoint because of mutations in p53. Therefore, the inhibition of Chk1 represents a strategy to increase the therapeutic efficacy of anticancer drugs by enhancing the apoptosis in tumour cells with a defective p53 response^12–15^. The first Chk1 inhibitor to enter phase I and II clinical trials against a wide range of tumour types was UCN-01 (7-hydroxystaurosporine)^16^^,^^17^. Many Chk1 inhibitors have subsequently been developed and subjected to phase I trials like AZD7762^18^, PF-477736^19^ and SCH900776^20^^,^^21^. Unfortunately, many of these clinical studies have been abandoned due to the low selectivity and the incidence of side effects of such compounds^15^^,^^22^.

**Scheme 1.**
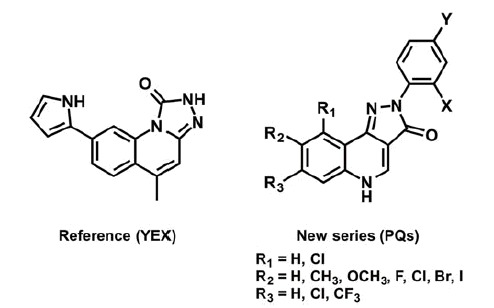
Reference structure (YEX) and PQs series studied.

In the search for new potent and selective Chk1 inhibitors, a congeneric series of 2-aryl-2 *H*-pyrazolo[4,3-*c*]quinolin-3-ones (PQs) was studied, for the first time. We present here the synthesis of a PQ library, containing 14 novel chemical entities, and the application of molecular modelling as supporting methodology to obtain preliminary data. Particularly, a hybrid approach based on docking/molecular dynamics (MD) simulations were implemented for the interpretation of the structure-activity relationship. For this purpose, a co-crystal Chk1 inhibitor (YEX, IC_50_ = 0.1 nM, and EC_50_ = 510 nM) was considered as reference compound since it has a similar PQ motif (Scheme 1)^10^^,^^11^.

In this preliminary study two compounds **4e_2_** and **4h_2_** have shown a modest, but encouraging, reduction in the basal activity of the Chk1 kinase. Starting from these encouraging results, we have designed the second generation of analogous in this class and further studies are in progress in our laboratories.

## Materials and methods

### General procedure for the conventional synthesis of PQ

For the conventional preparation of PQs, 0.15 g (0.44–0.69 mmol) of quinolones (**1a–h**) were mixed with thionyl chloride in excess (1 ml) at reflux temperature, for 1–2 h, to give the corresponding chloroquinolines (**2a–h**) in excellent yields (>95%). The excess of thionyl chloride was removed by evaporation and co-evaporation with dichloromethane (3 × 10 ml). The final products (**4a_1–2_, 4a_4_, 4b_2_, 4c_2_, 4d_1_, 4e_1_, 4e_3–4_, 4f_1–4_, 4g_4_, 4h_2–4_**) were obtained by reaction between **2a** and **h** with a 20% excess of the appropriated hydrazines **3_1–4_**: phenylhydrazine monohydrochloride, *o*-fluorophenylhydrazine monohydrochloride, *p-*fluorophenylhydrazine monohydrochloride and *p*-methoxyphenylhydrazine monohydrochloride. Hydrochloride salts were neutralised with equimolar amounts of triethylamine (TEA) before the addition of chloroquinolines. *N,N*-dimethylformamide (DMF) was used as solvent and the temperature of the process was kept between 130–140 °C, for 1–3.5 h as it is specified in Table 1. Finally, compounds **4b_2_**, **4d_1_**, **4e_1_**, **4f_1_**, and **4h_2–3_** were purified by recrystallisation from EtOH:H_2_O (96:4) and **4f_2–4_**, **4g_4,_** and **4h_4_** were purified by column chromatography using CHCl_3_: EtOH (9:1) as elution solvents. To purify **4e_3–4_** both methodologies were used, whereas **4a_1–2_**, **4a_4_** and **4c_2_** were obtained pure.

**Table 1.** Reaction times and yields for the synthesis of PQs 4a–h_1–4_

PQ	*R*_1_	*R*_2_	*R*_3_	X	Y	Heating method	Step1 (min)	Step2 (min)	Yield (%)^b^
4a_1_	H	H	H	H	H	C	60	120	70
4a_2_	H	H	H	F	H	C	60	90	78
4a_3_^a^	H	H	H	H	F	MW	1	5	90
4a_4_	H	H	H	H	OCH_3_	C	60	120	63
4b_1_	H	CH_3_	H	H	H	MW	1	1	88
4b_2_^a^	H	CH_3_	H	F	H	C	60	210	79 (63)
4b_3_	H	CH_3_	H	H	F	MW	1	1	78 (60)
4b_4_	H	CH_3_	H	H	OCH_3_	MW	1	1	75 (69)
4c_1_	H	OCH_3_	H	H	H	MW	1	4	73
4c_2_	H	OCH_3_	H	F	H	C	60	180	73
4c_3_^a^	H	OCH_3_	H	H	F	MW	1	5	70 (42)
4c_4_^a^	H	OCH_3_	H	H	OCH_3_	MW	1	4	64 (38)
4d_1_	H	Cl	H	H	H	C	120	150	55 (36)
4d_2_^a^	H	Cl	H	F	H	MW	2	2	67 (40)
4d_3_^a^	H	Cl	H	H	F	MW	2	2	59 (31)
4d_4_	H	Cl	H	H	OCH_3_	MW	2	2	92 (56)
4e_1_	Cl	H	Cl	H	H	C	120	150	89 (71)
4e_2_^a^	Cl	H	Cl	F	H	MW	2	2	61 (35)
4e_3_	Cl	H	Cl	H	F	C	120	90	87 (40)
4e_4_	Cl	H	Cl	H	OCH_3_	C	120	90	85 (41)
4f_1_	H	Br	H	H	H	C	90	60	92 (73)
4f_2_^a^	H	Br	H	F	H	C	90	210	91 (66)
4f_3_	H	Br	H	H	F	C	90	180	90 (76)
4f_4_	H	Br	H	H	OCH_3_	C	90	150	90 (56)
4g_1_^a^	H	I	H	H	H	MW	4	5	56 (27)
4g_4_^a^	H	I	H	H	OCH_3_	C	120	120	53 (23)
4h_1_^a^	H	F	CF_3_	H	H	MW	3	5	57 (15)
4h_2_^a^	H	F	CF_3_	F	H	C	120	120	87 (55)
4h_3_^a^	H	F	CF_3_	H	F	C	120	120	95 (74)
4h_4_^a^	H	F	CF_3_	H	OCH_3_	C	120	150	72 (43)

a New chemical entities.

b Yield calculated from ethyl-quinolin-4-one-3-carboxylate moles. In brackets the yield of purified product.

### General procedure for the microwave assisted synthesis of PQ

A CEM Discovery monomode equipment was used for the preparation of PQs applying MW heating in closed vessels. A mixture of 0.15 g (0.44–0.69 mmol) of quinolones (**1a–e**, **g–h**) and thionyl chloride in excess (1 ml) was irradiated with a dynamic power method at 85 °C, for 1–4 min, to give the chloroquinolines (**2a–e, g–h**) in excellent yields (>95%). The thionyl chloride was removed in the same way as previously described for the conventional synthesis. The final products (**4a_3_, 4b_1_, 4b_3–4_, 4c_1_, 4c_3–4_, 4d_2–4_, 4e_2_, 4g_1_, 4h_1_**) were obtained by reaction between **2a–e, g–h** with a 20% excess of the appropriated hydrazine hydrochlorides **3_1–4_**, previously neutralised with TEA, using 2 ml of DMF as solvent. The mixture of reactants was irradiated with a fix power method (300 W) at 140 ± 10 °C, for 1–5 min, as it is specified in Table 1. Finally, compounds **4b_3_**, **4c_3–4_**, **4d_2–3_**, and **4e_2_** were purified by recrystallisation from EtOH:H_2_O (96:4); compounds **4b_4_**, **4d_4_**, **4g_1_** and **4h_1_** were purified by column chromatography using CHCl_3_: EtOH (9:1) as elution solvent, whereas compounds **4a_3_**, **4b_1_**, and **4c_1_** were obtained pure.

### Characterisation of products

*2-Phenyl-2 H-pyrazolo[4,3-c]quinolin-3(5 H)-one (**4a_1_**):* yellow solid; m.p.: 328–331 °C^23^; ^1 ^H-NMR (400 MHz, DMSO-d_6_): δ (ppm) 7.16 (t, 1 H, *J* = 7.3 Hz); 7.44 (t, 2 H, *J* = 8.0 Hz); 7.54 (t, 1 H, *J* = 7.2 Hz); 7.66 (t, 1 H, *J* = 7.7 Hz); 7.80 (t, 1 H, *J* = 6.4 Hz); 8.22 (d, 3 H, *J* = 8.4 Hz); 8.66 (s, 1 H).

*2-(2-Fluorophenyl)-2 H-pyrazolo[4,3-c]quinolin-3(5 H)-one (**4a_2_**):* yellow solid; m.p.: not informed^24^; ^1 ^H-NMR (400 MHz, DMSO-d_6_): *δ* (ppm) 7.28–7.47 (m, 3 H); 7.50–7.60 (m, 2 H); 7.63–7.74 (m, 2 H); 8.13 (d, 1 H, *J* = 7.9 Hz); 8.72 (s, 1 H); 12.80 (s, 1 H).

*2-(4-Fluorophenyl)-2 H-pyrazolo[4,3-c]quinolin-3(5 H)-one (**4a_3_**):* yellow solid; m.p.: >300 °C; ^1 ^H-NMR (400 MHz, DMSO-d_6_): *δ* (ppm) 7.29 (t, 2 H, *J* = 9.0 Hz); 7.56 (t, 1 H, *J* = 8.1 Hz); 7.66–7.73 (m, 2 H); 8.20–8.25 (m, 3 H); 8.74 (s, 1 H); 12.88 (s, 1 H); ^13 ^C-NMR (400 MHz, DMSO-d_6_): δ (ppm) 106.50; 115.64; 115.86; 119.14; 120.03; 120.92; 122.58; 126.93; 130.65; 135.96; 137.10; 139.90; 143.51; 157.90; 160.30; 161.93.

*2-(4-Methoxyphenyl)-2 H-pyrazolo[4,3-c]quinolin-3(5 H)-one (**4a_4_**):* yellow solid; m.p.: 268–270 °C^23^; ^1 ^H-NMR (400 MHz, DMSO-d_6_): *δ* (ppm) 3.78 (s, 3 H); 7.02 (d, 2 H, *J* = 9.2 Hz); 7.55 (t, 1 H, *J* = 8.0 Hz); 7.63–7.73 (m, 2 H); 8.08 (d, 2 H, *J* = 9.2 Hz); 8.21 (d, 1 H, *J* = 7.9 Hz); 8.70 (d, 1 H, *J* = 5.1 Hz); 12.80 (s, 1 H).

*8-Methyl-2-phenyl-2 H-pyrazolo[4,3-c]quinolin-3(5 H)-one (**4b_1_**):* yellow solid; m.p.: 364–366 °C^25^; ^1 ^H-NMR (400 MHz, DMSO-d_6_): *δ* (ppm) 2.48 (s, 3 H); 7.17 (t, 1 H, *J* = 7.3 Hz); 7.44 (t, 2 H, *J* = 7.6 Hz); 7.50 (dd, 1 H, *J* = 1.8 and 8.7 Hz); 7.62 (d, 1 H, *J*= 8.4 Hz); 8.03 (s, 1 H); 8.22 (d, 2 H, *J* = 8.2 Hz); 8.68 (s, 1 H); 12.77 (s, 1 H); ^13 ^C-NMR (400 MHz, DMSO-d_6_): *δ* (ppm) 21.34; 106.38; 119.10; 119.88; 122.07; 124.39; 129.13; 131.87; 133.96; 136.73; 139.29; 140.64; 143.41; 162.16.

*2-(2-Fluorophenyl)-8-methyl-2 H-pyrazolo[4,3-c]quinolin-3(5 H)-one (**4b_2_**):* yellow solid; m.p.: >300 °C; ^1 ^H-NMR (400 MHz, DMSO-d_6_): *δ* (ppm) 2.45 (s, 3 H); 7.29–7.45 (m, 3 H); 7.49 (dd, 1 H, *J* = 1.5 and 8.3 Hz); 7.55–7.62 (m, 2 H); 7.93 (s, 1 H); 8.68 (d, 1 H, *J* = 6.3 Hz); 12.74 (d, 1 H, *J* = 5.3 Hz); ^13 ^C-NMR (400 MHz, DMSO-d_6_): *δ* (ppm) 20.85; 104.27; 116.32; 116.52; 118.77; 119.34; 121.50; 124.50; 128.46; 128.78; 128.85; 131.27; 133.31; 136.18; 139.01; 143.46; 154.97; 157.47; 161.59.

*2-(4-Fluorophenyl)-8-methyl-2 H-pyrazolo[4,3-c]quinolin-3(5 H)-one (**4b_3_**):* yellow solid; m.p.: not informed^24^; ^1 ^H-NMR (400 MHz, DMSO-d_6_): *δ* (ppm) 2.48 (s, 3 H); 7.28 (t, 2 H, *J* = 8.8 Hz); 7.50 (dd, 1 H, *J* = 1.8 and 8.3 Hz); 7.62 (d, 1 H, *J* = 8.6 Hz); 8.02 (s, 1 H); 8.22–8.25 (m, 2 H); 8.71 (d, 1 H, *J* = 6.5 Hz); 12.82 (d, 1 H, *J* = 6.8 Hz); ^13 ^C-NMR (400 MHz, DMSO-d_6_): *δ* (ppm) 20.86; 105.72; 115.18; 115.40; 118.60; 119.44; 120.37; 121.57; 131.44; 133.47; 136.30; 136.64; 138.97; 142.96; 157.37; 159.77; 161.48.

*2-(4-Methoxyphenyl)-8-methyl-2 H-pyrazolo[4,3-c]quinolin-3(5 H)-one (**4b_4_**):* yellow solid; m.p.: not informed^24^; ^1 ^H-NMR (400 MHz, DMSO-d_6_): *δ* (ppm) 2.48 (s, 3 H); 3.78 (s, 3 H); 7.01 (d, 2 H, *J* = 9.3 Hz); 7.49 (dd, 1 H, *J* = 1.8 and 8.5 Hz); 7.61 (d, 1 H, *J* = 8.3 Hz); 8.01 (s, 1 H); 8.09 (d, 2 H, *J* = 8.3 Hz); 8.65 (s, 1 H); 12.72 (s, 1 H); ^13 ^C-NMR (400 MHz, DMSO-d_6_): *δ* (ppm) 20.87; 55.23; 105.96; 113.80; 118.68; 119.38; 120.28; 121.52; 131.22; 133.38; 133.65; 136.15; 138.61; 142.47; 155.83; 161.10.

*8-Methoxy-2-phenyl-2 H-pyrazolo[4,3-c]quinolin-3(5 H)-one (**4c_1_**):* yellow solid; m.p.: 317–320 °C^25^; ^1 ^H-NMR (400 MHz, DMSO-d_6_): *δ* (ppm) 3.92 (s, 3 H); 7.17 (t, 1 H, *J* = 7.4 Hz); 7.30 (dd, 1 H, *J* = 2.8 and 9.1 Hz); 7.44 (t, 2 H, *J* = 8.0 Hz); 7.59 (d, 1 H, *J* = 2.9 Hz); 7.67 (d, 1 H, *J* = 9.0 Hz); 8.23 (d, 2 H, *J* = 7.6 Hz); 8.66 (s, 1 H); 12.82 (s, 1 H).

*2-(2-Fluorophenyl)-8-methoxy-2 H-pyrazolo[4,3-c]quinolin-3(5 H)-one (**4c_2_**):* yellow solid; m.p.: >300 °C^3^; ^1 ^H-NMR (400 MHz, DMSO-d_6_): *δ* (ppm) 3.90 (s, 3 H); 7.28 (dd, 1 H, *J* = 3.0 and 9.3 Hz); 7.32–7.45 (m, 3 H); 7.49 (d, 1 H, *J* = 3.0 Hz); 7.58 (td, 1 H, *J* = 1.5 and 7.9 Hz); 7.67 (d, 1 H, *J* = 3.0 Hz); 8.67 (d, 1 H, *J* = 6.5 Hz); 12.80 (d, 1 H, *J* = 6.3 Hz); ^13 ^C-NMR (400 MHz, DMSO-d_6_): *δ* (ppm) 55.66; 102.46; 103.55; 116.32; 116.51; 119.62; 120.14; 121.25; 124.55; 126.83; 128.77; 129.06; 129.61; 138.18; 143.55; 155.14; 157.55; 157.64; 161.64.

*2-(4-Fluorophenyl)-8-methoxy-2 H-pyrazolo[4,3-c]quinolin-3(5 H)-one (**4c_3_**):* yellow solid; m.p.: >300 °C; ^1 ^H-NMR (400 MHz, DMSO-d_6_): *δ* (ppm) 3.92 (s, 3 H); 7.26–7.31 (m, 3 H); 7.58 (d, 1 H, *J* = 2.7 Hz); 7.68 (d, 1 H, *J* = 9.0 Hz); 8.23–8.26 (m, 2 H); 8.69 (d, 1 H, *J* = 6.6 Hz); 12.87 (d, 1 H, *J* = 6.3 Hz); ^13 ^C-NMR (400 MHz, DMSO-d_6_): *δ* (ppm) 55.69; 102.57; 105.02; 115.17; 115.39; 119.74; 119.97; 120.40; 120.49; 121.32; 129.77; 136.67; 138.05; 143.01; 157.41; 157.60; 159.80; 161.49.

*2-(4-Methoxyphenyl)-8-methoxy-2 H-pyrazolo[4,3-c]quinolin-3(5 H)-one (**4c_4_**):* yellow solid; m.p.: >300 °C; ^1 ^H-NMR (400 MHz, DMSO-d_6_): *δ* (ppm) 3.78 (s, 3 H); 3.92 (s, 3 H); 7.01 (d, 2 H, *J* = 9.2 Hz); 7.29 (dd, 1 H, *J* = 2.6 and 9.0 Hz); 7.57 (d, 1 H, *J* = 2.7 Hz); 7.68 (d, 1 H, *J* = 8.9 Hz); 8.10 (d, 2 H, *J* = 9.0 Hz); 8.63 (s, 1 H); ^13 ^C-NMR (400 MHz, DMSO-d_6_): δ (ppm) 55.71; 56.13; 103.02; 105.70; 114.25; 119.90; 120.53; 120.95; 121.73; 130.19; 134.15; 138.10; 143.02; 156.36; 157.98; 161.62.

*8-Chloro-2-phenyl-2 H-pyrazolo[4,3-c]quinolin-3(5 H)-one (**4d_1_**):* yellow solid; m.p.: 396–400 °C^25^; ^1 ^H-NMR (400 MHz, DMSO-d_6_): *δ* (ppm) 7.21 (t, 1 H, *J* = 7.4 Hz); 7.48 (t, 2 H, *J* = 7.7 Hz); 7.69 (d, 1 H, *J* = 8.8 Hz); 7.85 (dd, 1 H, *J* = 2.3 and 8.8 Hz); 8.23 (d, 2 H, *J* = 8.0 Hz); 8.35 (d, 1 H, *J* = 2.0 Hz); 8.79 (s, 1 H).

*2-(2-Fluorophenyl)-8-chloro-2 H-pyrazolo[4,3-c]quinolin-3(5 H)-one (**4d_2_**):* yellow solid; m.p.: >320 °C; ^1 ^H-NMR (400 MHz, DMSO-d_6_): *δ* (ppm) 7.39–7.56 (m, 3 H); 7.66 (t, 1 H, *J* = 7.8 Hz); 7.81 (d, 2 H, *J* = 1.7 Hz); 8.16 (d, 1 H, *J* = 1.8 Hz); 8.85 (s, 1 H); 12.48 (s, 1 H); ^13 ^C-NMR (400 MHz, DMSO-d_6_): δ (ppm) 105.21; 117.00; 120.61; 121.49; 122.09; 125.03; 129.00; 129.59; 130.60; 131.09; 134.59; 140.26; 143.01; 155.50; 158.00; 161.91.

*2-(4-Fluorophenyl)-8-chloro-2 H-pyrazolo[4,3-c]quinolin-3(5 H)-one (**4d_3_**):* yellow solid; m.p.: >320 °C; ^1 ^H-NMR (400 MHz, DMSO-d_6_): *δ* (ppm) 7.38 (t, 2 H, *J* = 8.8 Hz); 7.75 (d, 1 H, *J* = 8.7 Hz); 7.78–7.83 (m, 2 H); 8.29–8.33 (m, 2 H); 8.87 (s, 1 H); ^13 ^C-NMR (400 MHz, DMSO-d_6_): δ (ppm) 106.67; 115.70; 115.92; 120.46; 120.93; 121.01; 121.61; 122.23; 130.71; 131.18; 134.78; 136.92; 140.27; 142.52; 158.02; 160.41; 161.76.

*2-(4-Methoxyphenyl)-8-chloro-2 H-pyrazolo[4,3-c]quinolin-3(5 H)-one (**4d_4_**):* yellow solid; m.p.: 326–328 °C^26^; ^1 ^H-NMR (400 MHz, DMSO-d_6_): *δ* (ppm) 3.87 (s, 1 H); 7.11 (d, 2 H, *J* = 9.2 Hz); 7.77–7.86 (m, 3 H); 8.16 (d, 2 H, *J* = 9.0 Hz); 8.81 (s, 1 H).

*7,9-Dichloro-2-phenyl-2 H-pyrazolo[4,3-c]quinolin-3(5 H)-one (**4e_1_**):* yellow solid; m.p.: >310 °C^27^; ^1 ^H-NMR (400 MHz, DMSO-d_6_): *δ* (ppm) 7.19 (t, 1 H, *J* = 7.2 Hz); 7.45 (t, 2 H, *J* = 7.5 Hz); 7.70 (d, 1 H, *J* = 1.8 Hz); 7.75 (d, 1 H, *J* = 1.8 Hz); 8.21 (d, 2 H, *J* = 7.5 Hz); 8.77 (s, 1 H); 12.77 (s, 1 H); ^13 ^C-NMR (400 MHz, DMSO-d_6_): *δ* (ppm) 107.79; 116.03; 117.65; 118.61; 124.34; 127.34; 128.78; 131.44; 133.58; 138.11; 139.74; 139.81; 141.18; 160.94.

*7,9-Dichloro-2–(2-fluorophenyl)-2 H-pyrazolo[4,3-c]quinolin-3(5 H)-one (**4e_2_**):* yellow solid; m.p.: >310 °C; ^1 ^H-NMR (400 MHz, DMSO-d_6_): *δ* (ppm) 7.39–7.58 (m, 3 H); 7.66 (td, 1 H, *J* = 1.5 and 7.8 Hz); 7.76 (d, 1 H, *J* = 1.8); 7.81 (d, 1 H, *J* = 1.8 Hz); 8.85 (s, 1 H); ^13 ^C-NMR (400 MHz, DMSO-d_6_): δ (ppm) 105.35; 116.81; 117.01; 119.22; 120.96; 122.26; 124.54; 125.03; 128.98; 129.58; 133.30; 134.95; 140.34; 142.85; 155.49; 157.99; 161.92.

*7,9-Dichloro-2–(4-fluorophenyl)-2 H-pyrazolo[4,3-c]quinolin-3(5 H)-one (**4e_3_**):* yellow solid; m.p.: >320 °C^27^; ^1 ^H-NMR (400 MHz, DMSO-d_6_): *δ* (ppm) 7.30 (t, 2 H, *J* = 9.0 Hz); 7.68 (s, 1 H); 7.75 (s, 1 H); 8.22 (m, 2 H); 8.78 (s, 1 H); 12.88 (s, 1 H); ^13 ^C-NMR (400 MHz, DMSO-d_6_): *δ* (ppm) 107.59; 115.28; 115.50; 115.94; 117.64; 120.34; 127.32; 131.39; 133.55; 136.29; 138.05; 139.77; 141.14; 157.59; 159.99; 160.69.

*7,9-Dichloro-2–(4-methoxyphenyl)-2 H-pyrazolo[4,3-c]quinolin-3(5 H)-one (**4e_4_**):* yellow solid; m.p.: >320 °C^27^; ^1 ^H-NMR (400 MHz, DMSO-d_6_): *δ* (ppm) 3.78 (s, 3 H); 7.03 (d, 2 H, *J* = 9.0 Hz); 7.67 (d, 1 H, *J* = 1.8 Hz); 7.73 (d, 1 H, *J* = 1.8 Hz); 8.09 (d, 2 H, *J* = 8.8 Hz); 8.74 (s, 1 H); 12.83 (s, 1 H); ^13 ^C-NMR (400 MHz, DMSO-d_6_): *δ* (ppm) 55.26; 107.80; 113.91; 116.07; 117.73; 120.31; 127.22; 131.30; 133.31; 138.15; 139.63; 140.72; 156.12; 160.38.

*8-Bromo-2-phenyl-2 H-pyrazolo[4,3-c]quinolin-3(5 H)-one (**4f_1_**):* yellow solid; m.p.: >305 °C^27^; ^1 ^H-NMR (400 MHz, DMSO-d_6_): *δ* (ppm) 7.18 (t, 1 H, *J* = 7.5 Hz); 7.45 (t, 2 H, *J* = 7.7 Hz); 7.66 (d, 1 H, *J* = 8.8 Hz); 7.83 (dd, 1 H, *J* = 2.3 and 8.8 Hz); 8.21 (d, 2 H, *J* = 8.0 Hz); 8.31 (d, 1 H, *J* = 2.0 Hz); 8.76 (s, 1 H); 12.89 (s, 1 H); ^13 ^C-NMR (400 MHz, DMSO-d_6_): *δ* (ppm) 106.49; 118.6; 118.68; 120.35; 121.80; 124.18; 128.68; 132.91; 134.56; 139.66; 139.93; 141.80; 161.47.

*8-Bromo-2-(2-fluorophenyl)-2 H-pyrazolo[4,3-c]quinolin-3(5 H)-one (**4f_2_**):* yellow solid; m.p.: >320 °C; ^1 ^H-NMR (400 MHz, DMSO-d_6_): *δ* (ppm) 7.31–7.47 (m, 3 H); 7.58 (td, 1 H, *J* = 1.6 and 7.7 Hz); 7.66 (d, 1 H, *J* = 8.8 Hz); 7.82 (dd, 1 H, *J* = 2.2 and 8.8 Hz); 8.20 (d, 1 H, *J* = 2.2 Hz); 8.76 (s, 1 H); 12.87 (s, 1 H); ^13 ^C-NMR (400 MHz, DMSO-d_6_): δ (ppm) 105.34; 116.81; 117.01; 119.21; 120.97; 122.28; 124.54; 124.99; 128.99; 129.57; 133.29; 134.98; 140.37; 142.85; 155.49; 157.98; 161.91.

*8-Bromo-2-(4-fluorophenyl)-2 H-pyrazolo[4,3-c]quinolin-3(5 H)-one (**4f_3_**):* yellow solid; m.p.: >365 °C^27^; ^1 ^H-NMR (400 MHz, DMSO-d_6_): *δ* (ppm) 7.29 (t, 2 H, *J* = 9.0 Hz); 7.67 (d, 1 H, *J* = 8.8 Hz); 7.84 (dd, 1 H, *J* = 2.3 and 8.8 Hz); 8.21–8.25 (m, 2 H); 8.31 (d, 1 H, *J* = 2.2 Hz); 8.79 (d, 1 H, *J* = 6.3 Hz); 12.94 (d, 1 H, *J* = 5.0 Hz); ^13 ^C-NMR (400 MHz, DMSO-d_6_): *δ* (ppm) 106.32; 115.25; 115.47; 118.88; 120.51; 121.85; 124.20; 132.99; 134.55; 136.41; 139.86; 141.85; 157.53; 159.96; 161.29.

*8-Bromo-2-(4-methoxyphenyl)-2 H-pyrazolo[4,3-c]quinolin-3(5 H)-one (**4f_4_**):* yellow solid; m.p.: >305 °C^27^; ^1 ^H-NMR (400 MHz, DMSO-d_6_): *δ* (ppm) 3.78 (s, 3 H); 7.01 (d, 2 H, *J* = 9.0 Hz); 7.65 (d, 1 H, *J* = 8.8 Hz); 7.81 (dd, 1 H, *J* = 2.0 and 8.8 Hz); 8.08 (d, 2 H, *J* = 9.0 Hz); 8.28 (d, 1 H, *J* = 2.0 Hz); 8.75 (s, 1 H); 12.87 (s, 1 H); ^13 ^C-NMR (400 MHz, DMSO-d_6_): *δ* (ppm) 55.26; 106.54; 113.85; 118.75; 120.43; 121.88; 124.13; 132.74; 133.42; 134.55; 139.59; 141.39; 156.02; 160.94.

*8-Iodo-2-phenyl-2 H-pyrazolo[4,3-c]quinolin-3(5 H)-one (**4g_1_**):* yellow solid; m.p.: >310 °C; ^1 ^H-NMR (400 MHz, DMSO-d_6_): *δ* (ppm) 7.12 (d, 3 H, *J* = 7.3 Hz); 7.46 (d, 1 H, *J* = 8.9 Hz); 7.82 (d, 1 H, *J* = 8.7 Hz); 7.93 (dd, 2 H, *J* = 1.7 and 8.6 Hz); 8.47 (d, 1 H, *J* = 1.5 Hz); 9.08 (s, 1 H).

*8-Iodo-2-(4-methoxyphenyl)-2 H-pyrazolo[4,3-c]quinolin-3(5 H)-one (**4g_4_**):* yellow solid; m.p.: >310 °C; ^1 ^H-NMR (400 MHz, DMSO-d_6_): *δ* (ppm) 3.77 (s, 3 H); 7.00 (d, 2 H, *J* = 9.0 Hz); 7.49 (d, 1 H, *J* = 8.5 Hz); 7.94 (dd, 1 H, *J* = 1.9 and 8.7 Hz); 8.07 (d, 2 H, *J* = 9.3 Hz); 8.47 (d, 1 H, *J* = 2.0 Hz); 8.72 (s, 1 H); 12.83 (s, 1 H); ^13 ^C-NMR (400 MHz, DMSO-d_6_): *δ* (ppm) 55.72; 91.85; 107.18; 114.31; 121.04; 122.08; 130.68; 133.89; 135.29; 138.72; 139.91; 141.56; 156.49; 161.43.

*8-Fluoro-2-phenyl-7-(trifluoromethyl)-2 H-pyrazolo[4,3-c]quinolin-3(5 H)-one (**4h_1_**):* yellow solid; m.p.: >300 °C; ^1 ^H-NMR (400 MHz, DMSO-d_6_): *δ* (ppm) 7.13 (d, 2 H, *J* = 7.2 Hz); 7.23–7.26 (m, 1 H); 7.30 (t, 2 H, *J* = 7.6 Hz); 8.28 (s, 1 H); 8.45 (d, 1 H, *J* = 7.1 Hz); 9.19 (s, 1 H).

*8-Fluoro-2–(2-fluorophenyl)-7-(trifluoromethyl)-2 H-pyrazolo[4,3-c]quinolin-3 (5 H)-one (**4h_2_**):* yellow solid; m.p.: >300 °C; ^1 ^H-NMR (400 MHz, DMSO-d_6_): *δ* (ppm) 7.34 (td, 1 H, *J* = 2.0 and 7.7 Hz); 7.40 (td, 1 H, *J* = 1.4 and 9.3 Hz); 7.44–7.50 (m, 1 H); 7.58 (td, 1 H, *J* = 1.7 and 7.7); 8.10 (m, 1 H); 8.13 (s, 1 H); 8.88 (s, 1 H); 13.0 (s, 1 H); ^13 ^C-NMR (400 MHz, DMSO-d_6_): *δ* (ppm) 104.98; 109.87; 110.09; 116.84; 117.03; 119.96; 124.26; 125.06; 129.10; 129.91; 132.21; 141.09; 142.62; 155.56; 158.05; 161.85.

*8-Fluoro-2-(4-fluorophenyl)-7-(trifluoromethyl)-2 H-pyrazolo[4,3-c]quinolin-3 (5 H)-one (**4h_3_***): yellow solid; m.p.: >300 °C; ^1 ^H-NMR (400 MHz, DMSO-d_6_): *δ* (ppm) 7.19 (t, 2 H, *J* = 8.9 Hz); 8.08 (d, 1 H, *J* = 6.27 Hz); 8.14 (s, 1 H); 8.16–8.20 (m, 2 H); 8.87 (s, 1 H); 13.02 (s, 1 H); ^13 ^C-NMR (400 MHz, DMSO-d_6_): *δ* (ppm) 106.42; 109.84; 110.06; 115.70; 115.92; 119.97; 121.10; 123.89; 132.19; 136.64; 140.86; 141.99; 158.16; 160.56; 161.60.

*8-Fluoro-2-(4-methoxyphenyl)-7-(trifluoromethyl)-2 H-pyrazolo[4,3-c]quinolin-3 (5 H)-one (**4h_4_**):* yellow solid; m.p.: >300 °C; ^1 ^H-NMR (400 MHz, DMSO-d_6_): *δ* (ppm) 3.79 (s, 3 H); 7.03 (d, 2 H, *J* = 8.9 Hz); 8.03–8.13 (m, 3 H); 8.18 (d, 1 H, *J* = 10.6 Hz); 8.87 (s, 1 H).

### Protein kinase assay

The inhibitory activity of PQ was carried out by ProQinase company (Freiburg, Germany). A radiometric protein kinase assay (^33^PanQinase^®^ Activity Assay) was used for measuring the kinase activity of the Chk1 protein kinase. All kinase assays were performed in 96-well FlashPlates^TM^ from Perkin Elmer (Boston, MA) in a 50 μl reaction volume. The reaction cocktail was pipetted in four steps in the following order: 10 μl of non-radioactive ATP aqueous solution (1 μM), 25 μl of assay buffer/[γ-^33 ^P]-ATP mixture, 5 μl of the test sample in 10% DMSO and 10 μl of the enzyme (25 ng/50 μL)/substrate (2 μg/50 μL) mixture. The assay for Chk1 protein kinases contained 70 mM HEPES-NaOH pH 7.5, 3 mM MgCl_2_, 3 mM MnCl_2_, 3 μM Na-orthovanadate, 1.2 mM DTT, ATP (variable amounts, corresponding to the apparent ATP-K_m_ of the respective kinase, [γ-^33 ^P]-ATP (approx. 8 × 10^5^ cpm per well). The final DMSO concentration in all reaction cocktails (including high and low controls) was 1%.

All protein kinases provided by ProQinase were expressed in Sf9 insect cells or in *Escherichia coli* as recombinant GST-fusion proteins or His-tagged proteins, either as full-length or enzymatically active fragments. All kinases were produced from human cDNAs. Kinases were purified by either GSH-affinity chromatography or immobilised metal. Affinity tags were removed from a number of kinases during purification. The purity of the protein kinases was examined by SDS-PAGE/Coomassie staining, the identity was checked by mass spectroscopy.

The reaction cocktails were incubated at 30 °C for 60 min. The reaction was stopped with 50 μl of H_3_PO_4_ (2% v/v), plates were aspirated and washed two times with 200 μl of aqueous NaCl (0.9% w/v). Incorporation of ^33^P_i_ (counting of “cpm”) was determined with a microplate scintillation counter (Microbeta, Wallac). All assays were performed with a BeckmanCoulter Biomek 2000/SL robotic system.

For each assay, the median value of the cpm of three wells with complete reaction cocktails, but without kinase, was defined as “low control” (*n* = 3). This value reflects unspecific binding of radioactivity to the plate in the absence of protein kinase but in the presence of the substrate. In addition, for each assay the median value of the cpm of three other wells with the complete reaction cocktail, but without any compound, was taken as the “high control”, i.e. full activity in the absence of any inhibitor (*n* = 3). The difference between high and low control was taken as 100% of the kinase activity. As part of the data evaluation, the low control value was subtracted from the high control value as well as from their corresponding “compound values”. The inhibition percentage for each compound was calculated by using the following formula:

$$Res.\;activity\;\left(\%\right)=\frac{\left(cpm\;of\;compound-low\;control\right)}{\left(high\;control-low\;control\right)}\times 100$$

$$Inhibition\;\left(\%\right)=100\%-Res.\;activity\;(\%)$$

### Computational simulations

*General.* All computations were performed on a hybrid CPU/GPU cluster. In particular, molecular docking simulations have been carried out using 8 Intel Xeon E5620 CPU cluster, whereas MD simulations were carried out with the ACEMD engine on a GPU cluster equipped with four NVIDIA GTX 580, two NVIDIA GTX 680, three NVIDIA GTX 780, and four NVIDIA GTX 980. The GOLD 5.2 suite^28^ was used for docking runs and a combination of protocols based on AMBER14^29^/general Amber force field (GAFF)^30^ was adopted for MD simulations. The Molecular Operating Environment (MOE, version 2014.09) suite^31^ and VMD 1.9^32^ were used for the visualisation analysis.

*Protein preparation.* The protein crystal structure PDB code 1ZYS^33^ was retrieved from the RCSB PDB database^34^. Crystallisation solvent and ions were removed, whereas hydrogen atoms were added, and appropriate ionisation states were assigned with the “Protonate-3D” tool^35^, as implemented in MOE 2014.09 suite^31^. Missing residues were modelled by the default homology modelling protocol implemented in the MOE protein preparation tool. Non-natural N-terminal and C-terminal were capped to mimic the previous residue. Then, the structure was subjected to energy minimisation with Amber99 force field^29^, by keeping the heavy atoms fixed at their positions. Finally, co-crystallised ligand and water molecules were removed and protein atoms partial charges computed with the Amber99 force field^29^.

*Ligands preparation.* Co-crystallised ligand YEX was extracted from the corresponding crystallographic complex (PDB code 2YEX)^11^, whereas all PQ were created with MOE 2014.09^31^. Hydrogen atoms were added to YEX, the protonation state (pH: 7.4) were assigned and they were subjected to a minimisation with MMFF94 force field^36^. For docking simulations, partial charges on ligands atoms were computed on the basis of the PM3/ESP semiempirical Hamiltonian^37^^,^^38^. Particularly, for post-docking MD simulations the ligands **4h_2_** and YEX were kept in their docking and crystallographic pose, respectively; whereas for supervised molecular dynamics (supervised molecular dynamics (SuMD) simulations, the ligands were subjected to two energy minimisation steps with MOPAC2012^39^ using PM6 method^40^ and Gaussian09^41^ (HF/6–31 G*). Then, ligand parameters were derived with GAFF(30) as implemented in Ambertools2014^29^ by using antechamber and paramcheck tools. RESP partial charges were calculated with Gaussian 09^41^ following the procedure suggested by antechamber. Only for SuMD simulations, ligands were placed at ∼30 Å from the mass centre of Chk1 ATP-binding site.

*Docking simulations.* Ligands were docked into the orthosteric binding site of the Chk1 protein with GOLD 5.2 suite^28^ using the GoldScore scoring function. The binding cavity centre was defined by the YEX ligand mass centre in the X-ray structure and the radius of such cavity set to 20 Å. For each docking run, 20 conformations were generated with an RMSD threshold 1.0 Å.

To estimate the electrostatic contributions to the binding energy of individual amino acids in the “interaction energy fingerprints” (IEFs) analysis, atomic charges for the ligands were computed on the basis of the PM3/ESP methodology, whereas partial charges for the protein amino acids were computed with the AMBER99 force field. The hydrophobic contributions have been calculated by using the directional hydrophobic interaction term based on contact surfaces as implemented in the MOE^31^.

*MD simulations – solvated system setup and equilibration.* Protein–ligand complexes were assembled with the tleap tool using AMBER14SB^42^ as the force field for the protein. The systems were explicitly solvated by a cubic water box using TIP3P as the water model^43^. To neutralise the total charge, Na^+^/Cl^−^ counterions were added to a final salt concentration of 0.154 M. The systems were energy minimised by 2500 steps with the conjugate-gradient method and then, they were subjected to two consecutive cycles of equilibration steps. Both cycles consisted of 25,000 steps of NVE (50 ps) followed by 500,000 steps of NPT (1 ns) simulation, using 2 fs as the time step. For the first cycle, a harmonic positional restraint of 1 kcal mol^−1^ Å^−2^ was applied on protein and ligand atoms, whereas for the second cycle, the restraint was applied only to the protein backbone. The pressure was maintained at 1 atm using a Berendsen barostat^44^. The Langevin thermostat was set with a low damping constant of 1ps^−145^. Bond lengths involving hydrogen atoms were constrained using the M-SHAKE algorithm^46^. The MD productive runs were conducted in an NPT ensemble. Long-range Coulomb interactions were handled using the particle mesh Ewald summation method setting the mesh spacing to 1.0 Å^47^.

SuMD is a command line tool written in Python, TCL, and Bash that operates the supervision of MD trajectories according to the algorithm that has been previously described by Cuzzolin et al.^48^ Three replicas were carried out for each ligand using the above-mentioned methodology. The production step of this kind of simulations depends on the time required for the ligand to reach the binding site (∼18–36 ns). On the contrary, the production step for post-docking MD was 100 ns.

## Results and discussion

### Synthesis of PQ

**Scheme 2.**
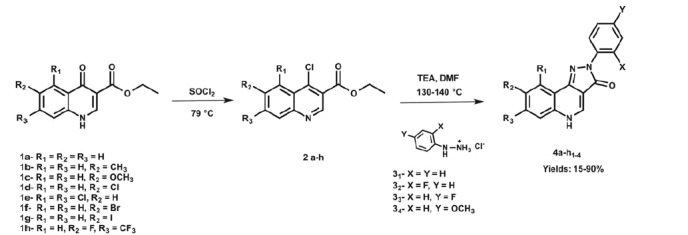
Synthesis of 2-aryl-2 *H*-pyrazolo[4,3-*c*]quinolin-3-ones from ethyl-quinolin-4-one-3-carboxylates.

A set of 2-aryl-2 *H*-pyrazolo[4,3-*c*]quinolin-3-ones (PQs) was synthesised from ethyl-quinolin-4-one-3-carboxylates using a protocol previously published by our research group^49^. The synthesis was carried out by using conventional (C) or microwave (MW) heating upon convenience. The López-Rivilli methodology^27^ was used for the former, whereas an analogous procedure was optimised for the MW-assisted synthesis of such compounds (Scheme 2). Table 1 summarises the conditions applied as well as the yield obtained for each compound.

Generally, the chlorination step (Step1) was quantitative for all the reactions (>95%), meaning that the pyrazolone ring formation (Step2) had more influence in the global yields of the reaction (15–90%). Even if the results obtained using MW heating were comparable to those where the synthesis was performed with conventional heating, the advantage of the first one was the reduction of reaction times from hours to minutes. To the best of our knowledge, the MW-assisted methodology was applied for the first time in the synthesis of such compounds. Moreover, it is important to highlight that 14 of the synthesised structures are new molecular entities.

### Docking simulations

As mentioned earlier, the synthesised PQs share a similar scaffold with some recently published Chk1 inhibitors^9^^,^^11^^,^^50^. Encouraged by the low IC_50_ value of these reference structures (980–0.1 nM), we decided to give our compounds a chance to be tested as Chk1 inhibitors.

In order to select the most appropriate analogous to perform the biological assay, docking simulations of the synthesised PQs were carried out using the scoring function *goldscore,* implemented in GOLD Suite 5.2^28^, and the Chk1 crystal structure 1ZYS^33^^,^^51^, which were previously validated by using DockBench 1.0^52^.

Although there are three tautomeric forms possible for such PQs, only the most stable tautomer in solution (keto tautomer shown in Scheme 1) was considered for the docking study^53^. The selection of the most favourable pose of each compound investigated was carried out by computing the total electrostatic and hydrophobic contributions of each pose to the interaction energy (IE). Moreover, the hydrogen bond interactions with Chk1 binding site residues and the IE patterns, displayed as 3 D colour maps, were also considered for the selection (data not shown).

**Figure 1.**
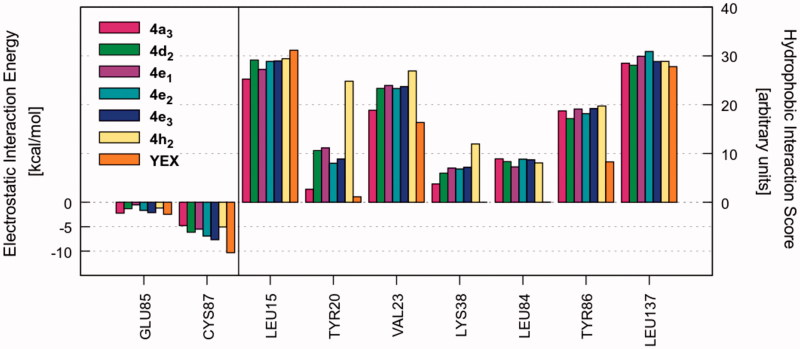
IEFs of **4a_3_, 4d_2_, 4e_1–3_, 4h_2_** and reference YEX. The key residues involved in the binding are displayed (*x* values) along with the electrostatic interaction energy (left) and the hydrophobic interaction score (right).

Once the most favourable pose of each compound was identified, the ligand–receptor interaction was analyzed in a more quantitative manner. That is, we calculated the individual electrostatic and hydrophobic contributions to the IE of each receptor residue involved in the binding with the ligand. The analysis of these contributions has been reported as IEFs, showing the key residues involved in the binding with the considered ligands along with a quantitative estimate of the occurring interactions (Video S1). Figure 1 displays the IEFs for the six PQs with the most favourable interactions with Chk1 binding site residues, as well as a comparison with the reference inhibitor YEX.

**Figure 2.**
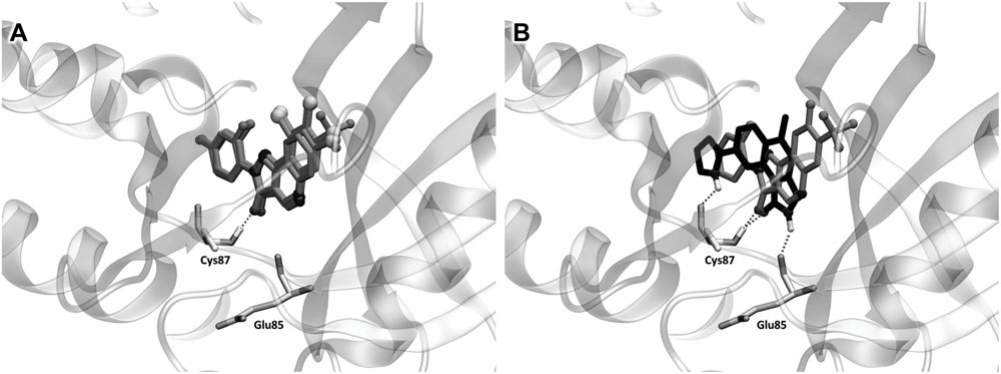
(A) Binding mode of PQs into the Chk1. (B) Comparison of the ligands binding modes between PQ **4h_2_** (grey) and reference YEX (black). The hydrogen bond interactions are shown as dashed lines.

The most important residues involved in the hydrophobic interactions between the Chk1 binding site and the studied ligands are Leu15, Val23, Tyr86, and Leu137. Particularly, compound **4h_2_** also shows a strong interaction with Tyr20 (Figure 1, right panel). Regarding the electrostatic interactions, Glu85 and Cys87 are the residues with the higher contribution, being also the most frequently involved residues in hydrogen bond interactions with the kinase inhibitors^54–57^. For the case of the investigated PQs, which share a common binding mode (Figure 2(A)), the electrostatic interaction with Cys87 is more favourable than Glu85 (Figure 1, left panel). In addition, the amide nitrogen of Cys87 establishes a hydrogen bond with the carbonyl oxygen of pyrazolone ring. Such interaction is also observed with the triazolone ring of the reference compound YEX, although the binding mode, and therefore, the hydrogen bond interaction profile is different (Figure 2(B)). The X-ray crystal structure of YEX shows three hydrogen bonds: the above-described between the carbonyl oxygen of triazolone and Cys87, another one between the pyrrole nitrogen and Cys87 and the third one between the triazolone nitrogen and the amide carbonyl of residue Glu85.

### Chk1 inhibitory activity

In order to have a first idea about the kinase inhibition profile of the studied PQs, a Chk1 protein kinase assay at one concentration (1 μM) was carried out for a dozen of compounds. At the same time, MD simulations were performed in order to understand the importance of the binding interactions profile at the molecular level.

For the kinase inhibition test, we selected not only the structures shown in Figure 1 but also some analogous compounds (maintaining the quinolone substitution) with a different type of substituents in the phenylpyrazole ring (i.e. **4d_3–4_**, **4e_4_**, and **4h_3_**). Moreover, two derivatives which shown unfavourable energy interactions into the binding site were included (i.e. **4c_3_** and **4f_3_**). Such selection was made with the aim to correlate the potential inhibitory activity of the investigated PQs according to their substituents. Unfortunately, the profiling of the tested compounds (**4a_3_**, **4c_3_**, **4d_2–4_**, **4e_1_, 4e_3–4_**, **4f_3_**, **4h_3_**) against Chk1 protein kinase showed no significant inhibitory potency at an assay concentration of 1 μM with the exception of the two compounds **4e_2_** and **4h_2_** which show a modest but significant (approximately 10%) reduction in the basal activity of the kinase (Table 2).

**Table 2.** Residual activity and inhibition percentages of Chk1 protein kinase induced by PQs.

PQ	*R*_1_	*R*_2_	*R*_3_	X	Y	Residual activity (%)^a^	Inhibition (%)
4a_3_	H	H	H	H	F	97	3
4c_3_	H	OCH_3_	H	H	F	121	0
4d_2_	H	Cl	H	F	H	96	4
4d_3_	H	Cl	H	H	F	110	0
4d_4_	H	Cl	H	H	OCH_3_	124	0
4e_1_	Cl	H	Cl	H	H	109	0
4e_2_	Cl	H	Cl	F	H	89	11
4e_3_	Cl	H	Cl	H	F	117	0
4e_4_	Cl	H	Cl	H	OCH_3_	123	0
4f_3_	H	Br	H	H	F	123	0
4h_2_	H	F	CF_3_	F	H	91	9
4h_3_	H	F	CF_3_	H	F	98	2

a Mean value of two independent analysis.

### MD simulations

Thus, these seemingly disappointing results offer a great opportunity to strengthen scientific investigation in this novel class of potential Chk1 inhibitors. Starting from the biological activity observed for the two compounds **4e_2_** and **4h_2_**, we decided to extent the molecular modelling simulations in order to rationalise the experimental results. We propose here to take advantage of MD simulations to figure out the possible causes of their low activity as Chk1 inhibitors, which could be useful for the design of new candidates.

**Figure 3.**
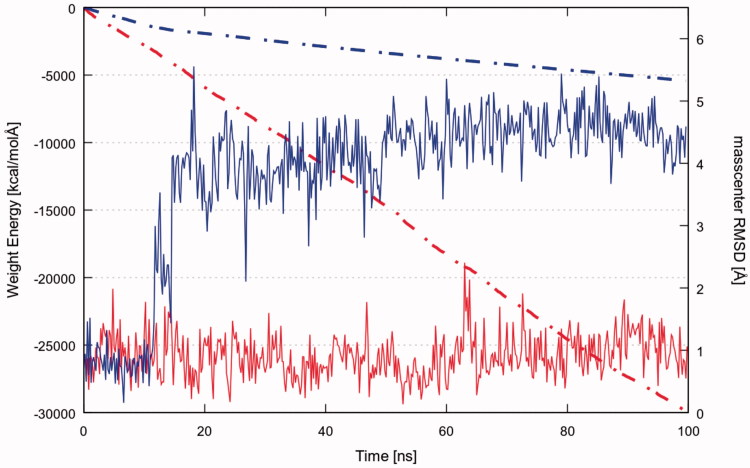
RMSD of **4h_2_** (blue continuous line) and YEX (red continuous line) with respect to its mass centre; and the cumulative sum of the total IE values for each frame against the time for **4h_2_** (blue dashed line) and YEX (red dashed line).

As a first attempt, we investigated the stability of the ligand–protein complexes through MD simulations of **4h_2_** (selected from previous docking simulations) and YEX (reference inhibitor)^10^^,^^11^ starting from its docking and crystallographic coordinates, respectively. The ligand RMSD plot shows that the docking pose of compound **4h_2_** is not stable since its coordinates are not retained during the simulation (Video S2). The mentioned ligand undergoes through two major changes in its orientation until reach a more stable conformation. The first orientation is retained for about 11 ns (RMSD ∼0.8 Å) and the second one is maintained until the end of the simulation with an average RMSD ∼4 Å (Figure 3, blue curve). The dashed line in Figure 3 shows the cumulative sum of the total IE values for each frame of the time. Hence, changes in the observed trend highlight how the variation of ligand conformation/position affects the IE. Therefore, the change in the slope of the cumulative ligand − protein IE plot (Figure 3, blue dashed line) suggests that the first position explored by **4h_2_** is energetically more favourable than the last one. On the other hand, YEX does not suffer large conformational changes (Video S3). Figure 3 shows that the reference ligand remains stable in its initial crystallographic position (RMSD oscillating around ∼1 Å) with constant IE since no changes are observed in the slope of the cumulative IE plot (red dashed line).

In particular, it is noteworthy to mention that the IE of YEX with the protein is much more favourable than the corresponding for PQ **4h_2_**, as it is apparent from the comparison of both cumulative IE plots (see Figure 3, dashed lines).

The higher stability of the YEX-Chk1 complex suggests a lower dissociation rate constant (*k*_off_) for this compound than for **4h_2_**. However, through the MD simulations performed it is not possible to get information about the binding recognition event, which is related to the association rate constant (*k*_on_). Therefore, in order to complete the qualitative thermodynamic analysis, the recently implemented methodology SuMD^48^ was used to compare the ligand–protein recognition pathway of both compounds.

**Figure 4.**
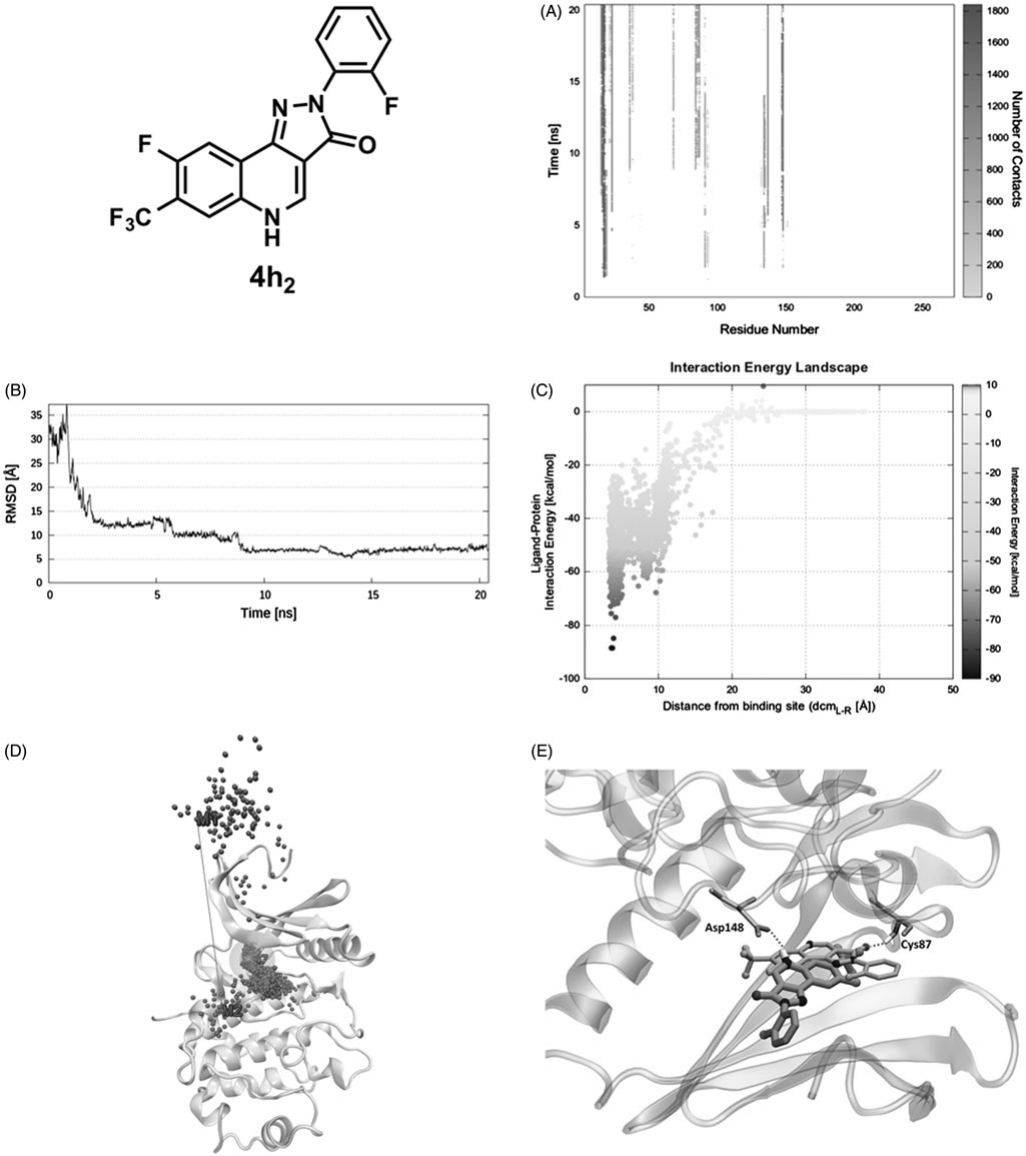
PQ **4h_2_** recognition pathway: (A) ligand − protein recognition map, (B) ligand − RMSD, (C) IE landscape, (D) Pollicino analysis (M1, M2, M3, and M4, indicate clusters) and (E) hydrogen bond interactions (dashed lines) of final SuMD pose in comparison with the pose suggested by docking simulation.

In the starting geometry, **4h_2_** was placed at a distance of 30 Å from the binding site. As depicted in Figure 4(A) and shown in Video S4, the first interaction between the ligand and the protein is established after 1.5 ns of productive trajectory and is mediated by Glu17, Gly18, and Ala19. Such residues move along with the ligand making new interactions with Glu91, Glu134, and Asp148, where it resides for about 2.7 ns. In fact, the ligand RMSD plot (Figure 4(B)) records stable values in the 2 − 4.7 ns time lapse. Afterward, guided by Lys132, Glu134, and Asp148, **4h_2_** is oriented to the binding site remaining there for about 3 ns (second plateau Figure 4(B)). The IE with the protein in this site is about −55 kcal/mol (Figure 4(C) at dcm_L-R_ = 8 Å). Approximately after 9 ns of simulation the ligand moves toward the orthosteric site, where Leu15 and Glu91 are the key residues that induce the ligand to find its final conformation. Consistently, the RMSD plot presents another plateau in the time range of 9 − 20 ns (Figure 4(B)) that corresponds to the swarm of dots in the IE landscape at dcm_L-R_ = 4 Å and around –50 and –70 kcal/mol (Figure 4(C)). During the last 10 ns of simulation, **4h_2_** interacts with Val23, Lys38, Leu 84, Glu85, Tyr86, Cys87, Glu91, Leu137, Ser147, and Asp148. The ligand–protein recognition map (Figure 4(A)) highlights that the above-mentioned residues are those establishing the greatest number of contacts, whereas the Pollicino analysis summarises the ligand recognition pathway (Figure 4(D)). Finally, it is important to mention that the final SuMD pose is different from that suggested by the docking simulation (RMSD ∼7 Å, Figure 4(B)) so that the hydrogen bond with Cys87 observed in the latter is not present in SuMD pose which instead interacts with Asp148 (Figure 4(E)).

**Figure 5.**
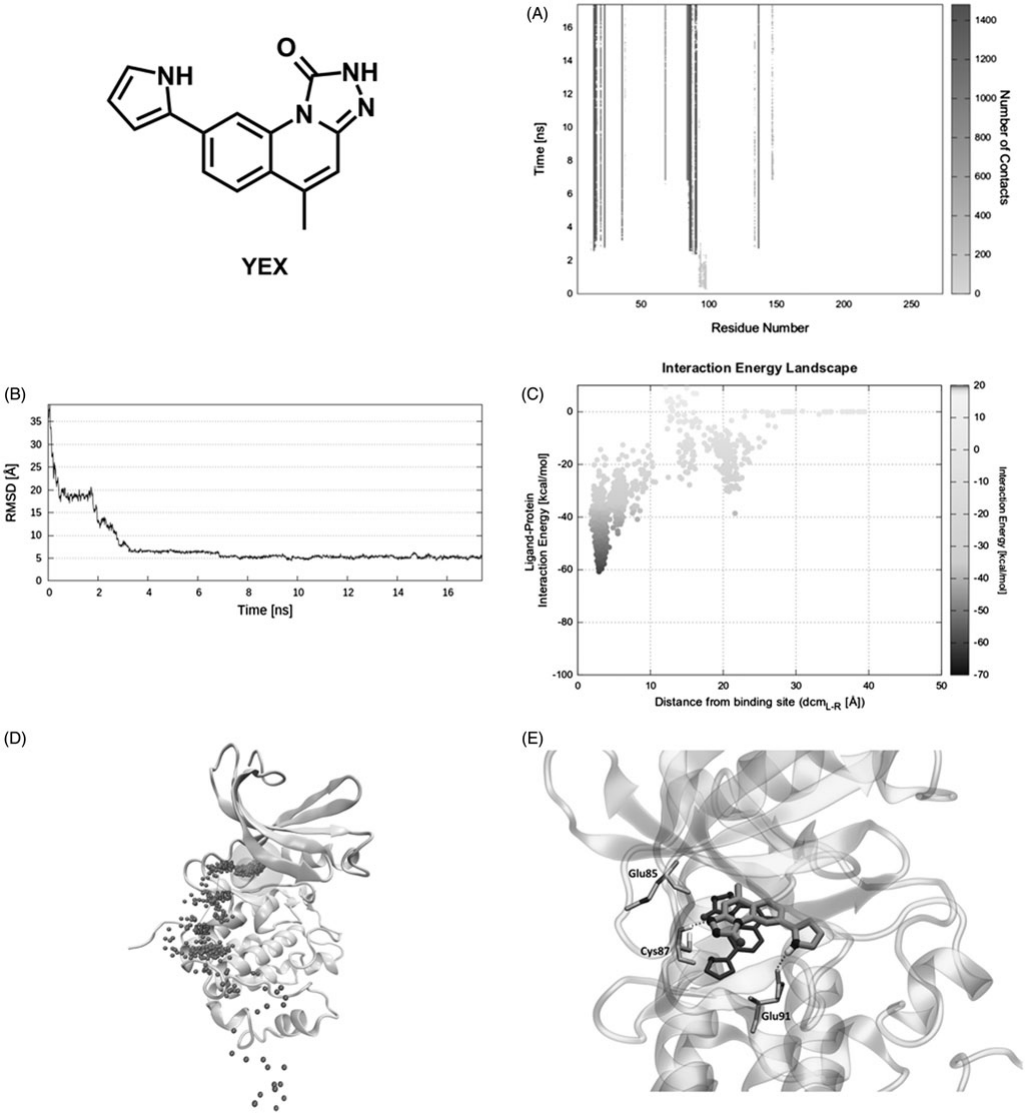
Reference compound YEX recognition pathway: (A) ligand − protein recognition map, (B) ligand − RMSD, (C) IE landscape, (D) Pollicino analysis (M1, M2, M3, M4, and M5 indicate clusters) and (E) hydrogen bond interactions (dashed lines) of final SuMD pose in comparison with the crystallographic pose.

On the other hand, the first interaction of the reference compound YEX with Chk1 is mediated by residues Glu97, Pro98, and Asp99 (Figure 5(A)). However, the ligand is immediately stabilised by nearby residues Asp94, Arg95, and Ile96 until 2 ns of productive trajectory (first plateau, Figure 5(B)). The latter three residues along with Gly89 and Gly90 induce a shift in the ligand position that places it toward the orthosteric site. Mediated by the same key residues (Glu91 and Leu15) found in the previous case, YEX reaches the binding site in less than 4 ns (second plateau, Figure 5(B)). The ligand–protein recognition map (Figure 5(A)) shows the ligand main contacts with residues Leu15, Gly16, Glu17, Val23, Lys38, Leu84, Glu85, Cys87, Glu91, Leu137, and Ser147 during the rest of SuMD simulation (Video S5). Meanwhile, the IE landscape shows that there is a unique zone explored by the ligand that corresponds to the highly populated region at dcm_L−R_ = 3 Å and around –40 and –60 kcal/mol (Figure 5(C)). The same conclusion arises from Pollicino analysis (Figure 5(D)) where the ligand pathway converges quickly in the binding site. Once YEX reaches the binding site, that pose is maintained until the end of the simulation (17.5 ns), with the exception of conformational changes occurring to the pyrrole ring, able to establish a hydrogen bond with the side chain of Glu91 (Figure 5(E)). Although the final SuMD pose seems to be close to the structure of the complex obtained by X-ray crystallography, the RMSD between them is ∼5 Å (Figure 5(B)) as they are rotated about 100°. Such conformation implies the loss of the hydrogen bond with Glu85 respect to the crystallographic structure (Figure 5(E)).

Whereas traditional MD simulations revealed an important difference in the stability of the complexes, SuMD analysis suggests that the *k*_on_ of both compounds are not as different as their *k*_off_. In other words, the PQ nucleus decoration would affect the dissociation but not the binding process. These observations indicate that the scaffold modification based on the final state interactions would result in new active compounds without affecting the binding process.

### Docking simulations of newly proposed compounds

Interestingly, the reference compound YEX is strongly trapped in the binding cavity since it retains three hydrogen bonds throughout the MD simulation. As it was described above, the triazolone ring is oriented in a way that allows the formation of two hydrogen bonds interactions: one between its carbonyl group and the amide nitrogen of residue Cys87, and the other between the triazole NH group and the carbonyl oxygen of residue Glu85. In addition to those interactions, the pyrrole ring present in YEX is a key portion of the formation of an additional hydrogen bond with the carbonyl of residue Cys87 (Figure 2(B)). It is important to highlight that the last mentioned moiety is essential for the inhibitory activity of YEX, as it was demonstrated by Lv et al.^57^

**Scheme 3.**
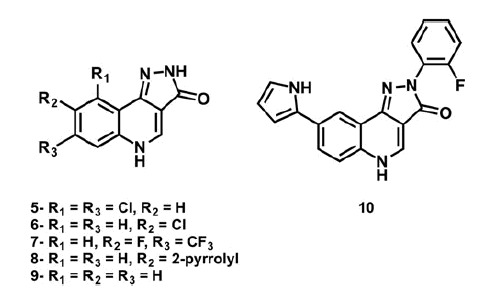
New proposed compounds **5–10**.

With these observations in mind, we propose some chemical modifications to perform on the PQ nucleus with the aim to improve the affinity of such compounds for Chk1 kinase. First, the removal of phenyl group from pyrazolone ring was applied to increase the possibilities of the N-H pyrazolone to establish hydrogen bonds (either as donor or acceptor) as it is present in YEX structure. Moreover, the addition of pyrrole ring to the quinolone scaffold and a combination of both alternatives were also taken into account (Scheme 3).

**Figure 6.**
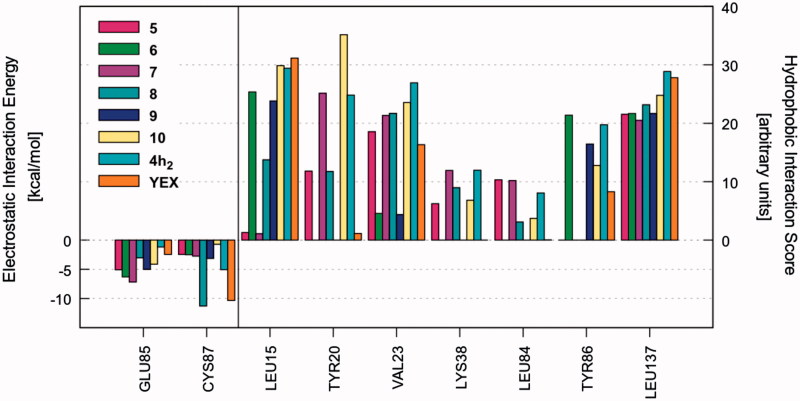
IEFs of new proposed compounds **5**–**10**. The interactions observed for **4h_2_** and YEX are also included for comparison.

The electrostatic and hydrophobic contributions to the IE of the newly proposed compounds are displayed in Figure 6 as IEFs (Video S6).

**Figure 7.**
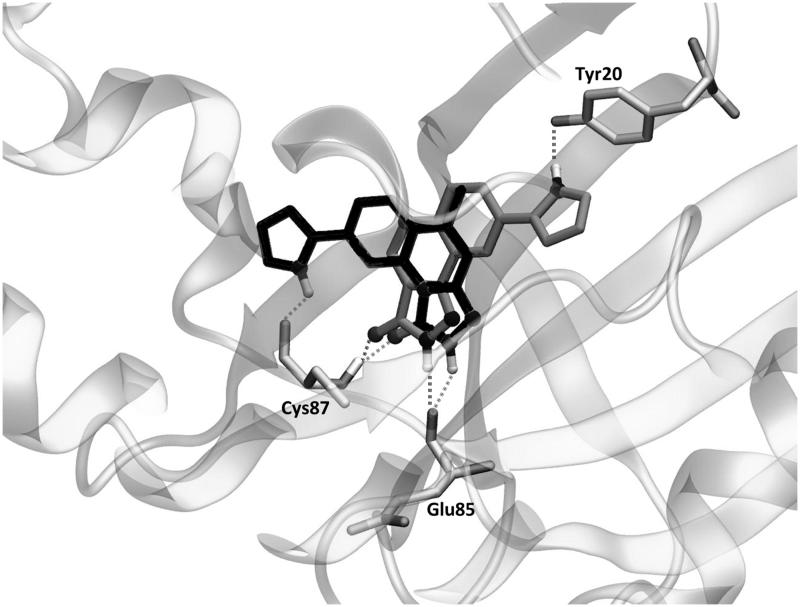
The binding mode of YEX (black) and new proposed compound 8 (grey) into the Chk1. Hydrogen bond interactions are shown as dashed lines.

A first glance at the docking poses of the new derivatives revealed that the exclusion of phenyl ring improves the electrostatic interaction with Glu85 but decreases the hydrophobic interactions with some of the receptor residues involved in the binding with the ligand. On the other hand, the simple addition of the pyrrole ring, by maintaining the phenyl group in the pyrazolone (compound **10**), does not show any improvement in the electrostatic contribution (Figure 6). Finally, the best results arose from compound **8**, which shares with YEX a common positioning of the pyrazolone ring. It is worth recalling that such orientation allows the formation of hydrogen bonds with Cys87 and Glu85 as it was mentioned above for YEX (Figure 7). Furthermore, the additional hydrogen bond interaction with Glu85 would not be possible when the pyrazolone moiety is substituted with a phenyl group which in that case, makes the ligand place with a different binding mode (Figure 2).

As is suggested in Figures 6 and 7, compound **8** has demonstrated not only an improvement in the electrostatic contribution to the IE with Cys87 but also an enhancement of the hydrogen bond interactions, essential for the inhibitory activity as some pharmacophore models propose^56^^,^^58^. Taking all these factors into account, a new library of PQs is being synthesised considering the modifications proposed and they will be tested as Chk1 inhibitors in the next future.

## Conclusions

A library of 2-aryl-2 *H*-pyrazolo[4,3-*c*]quinolin-3-ones, containing 14 new chemical structures, was synthesised using conventional or MW heating. The yields after purification were ranged from 15 to 90%, independently of the methodology used. However, the reaction times were considerably reduced when MW heating was applied.

For the first time, the selected PQs were tested as Chk1 inhibitors. Therefore, the implementation of molecular modelling, especially a hybrid approach based on docking and MD simulations, was critical for the understanding of the ligand–protein interactions at molecular level and, to generate a possible hypothesis to the experimentally observed inactivity of these compounds.

Starting from docking simulations, a difference in the binding mode was identified for the studied compounds with respect to the reference inhibitor YEX. Whereas, the PQs make only one hydrogen bond interaction with Cys87, the reference YEX has two interactions with Cys87 and one with Glu85. This hydrogen bond network of YEX is retained during the MD simulation, suggesting that a more stable complex with the protein is formed compared with **4h_2_**, which changed its conformation as soon as simulation starts. Moreover, the use of SuMD offered a precious insight into the ligand–protein recognition pathway. From the comparative analysis of both simulations, it is worthy to remark the role of residues Leu15 and Glu91 in the ligand entrance to the binding site. The SuMD analysis suggests that the entrance path is weakly dependent on the chemical substitution of the ligand. Thus, ligand affinity could be improved by changing the scaffold decoration without affecting the recognition pathway. Indeed, the results of MD simulations guided us to propose new structures by taking into account the hydrogen bond arrangement observed for YEX.

It is important to mention that one promising compound emerged from this study. The new candidate presents not only an improvement in the electrostatic contribution to the IE with Cys87 but also an enhancement of the hydrogen bond interactions, essential for the inhibitory activity.

Finally, we want to emphasise the importance of getting useful information from an apparently disappoint result, which could be the beginning for further hits identification. Besides, this article illustrates the relevance of using molecular modelling as part of an iterative cycle of design, synthesis, biological evaluation, hypothesis generation and leader optimisation in the discovery of new potential drug candidates.

## Supplementary Material

IENZ_1404592_Supplementary_Material.zip
